# Characterization of the TNFR1-d2 protein: Implication in TNF receptor associated periodic syndrome (TRAPS)?

**DOI:** 10.1186/1546-0096-13-S1-P39

**Published:** 2015-09-28

**Authors:** C Rittore, E Sanchez, S Soler, V Ea, D Genevieve, I Touitou, S Grandemange

**Affiliations:** 1 Laboratoire des maladies rares et auto-inflammatoires, Hopital A. de Villeneuve, Montpellier, France; 2 Inserm / Chu, U1183, Montpellier, France; 3 Département de génétique médicale, Hopital A. de Villeneuve, Montpellier, France; 4 CHU Caremeau, Pole psychiatrie, Nimes, France; 5 Institut de génétique moléculaire, Montpellier, France; 6 Université, Montpellier, France

## Introduction

Binding of TNF to TNF receptor 1 (TNFR1) induces both the survival pathway by activation of the NF-kB transcription factor, and the death pathway by apoptosis. Mutations in the TNFR1 gene (*TNFSFR1A*) are responsible for the auto-inflammatory disease TRAPS, a dominantly inherited hereditary recurrent fever. Various defects such as defective TNFR1 receptor shedding, protein misfolding, NF-kB activation, or apoptosis have been associated with the pathogenesis of TRAPS.

Previously, we have identified TNFR1-d2, an exon2-spliced transcript of *TNFRSF1A*. TNFR1-d2 is expressed in a tissue-specific manner in contrast to ubiquitous expression of the full-length TNFR1 transcript.

## Objectives

This study aimed to analyze the TNFR1-d2 protein expression and its function in NF-kB signalling pathways and to investigate the possible role of TNFR1-d2 in TRAPS physiopathology.

## Materials and methods

Translation analyses of TNFR1-d2 were performed in HEK293T by over-expression of different TNFR1-d2 cDNA constructs fused to the Flag tag. HEK293T transfected cells were used to measure internal ribosome entry site (IRES) activity and NF-κB-activation by luciferase assays. Subcellular localization of the TNFR1-d2 fused to the GFP protein was studied in MCF7 cells, followed by staining of different cellular compartments and confocal fluorescence microscopy analysis.

## Results

We showed that TNFR1-d2 is translated from an alternative start codon due to an IRES activity created by the exons 1 and 3 junction. The methionine 109 located in exon 4 in-frame with TNFR1 was used, resulting in a putative new protein isoform lacking its N-terminal region. Subcellular localization showed that the full-length and TNFR1-d2 proteins shared the same intracellular localization to the Golgi complex. Since the c.224C>T (p.Pro75Leu, P46L) and c.236C>T (p.Thr79Met, T50M) mutations in exon 3 lie in close vicinity to a strong Kozak consensus sequence, we hypothesized that these 2 sequence variants could affect TNFR1-d2 translation. Interestingly, we found that only TNFR1-d2 carrying the severe T50M mutation was translated through the mutated codon which induced a decrease of the IRES activity. Moreover, whereas overexpression of wild type TNFR1-d2 was not associated with increased NF-κB transcriptional activity, TNFR1-d2-T50M seemed to increase NF-κB activity as compared to the empty vector.

## Conclusion

Our results support that the TNFR1-d2-T50M translation defect could lead to a gain-of-function of TNFR1-d2, suggesting that TNFR1-d2 may account for the physiopathology of TRAPS in patients carrying the T50M mutation, which is associated with a severe TRAPS phenotype.

